# Commutators associated with Schrödinger operators on the nilpotent Lie group

**DOI:** 10.1186/s13660-017-1584-8

**Published:** 2017-12-19

**Authors:** Tianzhen Ni, Yu Liu

**Affiliations:** School of Mathematics and Physics, University of Science and Technology Beijing, Beijing, China

**Keywords:** 22E30, 42B35, 35J10, 42B20, commutator, Lipschitz space, nilpotent Lie groups, reverse Hölder inequality, Riesz transform, Schrödinger operator

## Abstract

Assume that *G* is a nilpotent Lie group. Denote by $L=-\Delta +W $ the Schrödinger operator on *G*, where Δ is the sub-Laplacian, the nonnegative potential *W* belongs to the reverse Hölder class $B_{q_{1}}$ for some $q_{1} \geq \frac{D}{2}$ and *D* is the dimension at infinity of *G*. Let $\mathcal{R}=\nabla (-\Delta +W)^{-\frac{1}{2}}$ be the Riesz transform associated with *L*. In this paper we obtain some estimates for the commutator $[h,\mathcal{R}]$ for $h\in \operatorname{Lip}^{\theta }_{\nu }$, where $\operatorname{Lip}^{\theta }_{\nu }$ is a function space which is larger than the classical Lipschitz space.

## Introduction

Assume *G* to be a connected and simply connected nilpotent Lie group and $\frak{g}$ to be its Lie algebra identified with the space of left invariant vector fields. Given $X=\{X_{1},\ldots ,X_{l}\}\subseteq \frak{g}$, a Hörmander system of left invariant vector fields on *G*. Let $\Delta =\sum^{l}_{i=1} X^{2}_{i}$ be the sub-Laplacian on *G* associated with *X* and the gradient operator ∇ be denoted by $\nabla = (X_{1}, \ldots , X_{l})$. Following [1], one can define a left invariant metric *d* associated with *X* which is called the Carnot-Carathéodory metric: let $x,y\in G$, and

$$\begin{aligned} d(x,y)=\inf \bigl\{ \delta \mid \exists \gamma \colon [0,\delta ]\rightarrow G \mid \gamma (0)=x,\gamma (\delta )=y \bigr\} , \end{aligned}$$

where *γ* is a piecewise smooth curve satisfying

$$ \gamma '(s)=\sum^{l}_{i=1}a_{i}(s)X_{i} \bigl(\gamma (s)\bigr), \quad \forall s\in [0,\delta ], \sum ^{l}_{i=1}\bigl\vert a_{i}(s) \bigr\vert ^{2}\leq 1. $$

If $x\in G$ and $r>0$, we will denote by $B(x,r)=\{y\in G \mid d(x,y)< r \}$ the metric balls. Assume d*x* to be the Haar measure on *G*. Then, for every measurable set $E\subseteq G$, $\vert E \vert $ denotes the measure of *E*. Suppose *e* to be the unit element of *G*. Note that $V(r)=\vert B(e,r) \vert =\vert B(x,r) \vert $ for any $x\in G$ and $r>0$. It follows from in [2] or [3] that there exists a constant $C_{1}>0$ such that

1 $$\begin{aligned}& C^{-1}_{1}r^{d} \leq V(r)\leq C_{1} r^{d}, \quad \forall 0\leq r\leq 1; \end{aligned}$$

2 $$\begin{aligned}& C^{-1}_{1}r^{D}\leq V(r)\leq C_{1} r^{D},\quad \forall 1\leq r< \infty, \end{aligned}$$

where *d* and *D* denote the local dimension and the dimension at infinity of *G*, and there is $D\geq d>0$. At this time, the Lie group *G* is also called a Lie group of polynomial growth. If *G* is a stratified Lie group, then $D= d$ (cf. [1]). Also, there exist positive constants $C_{2}, C_{3}>1$ such that

3 $$\begin{aligned}& C^{-1}_{2}\biggl(\frac{R}{r} \biggr)^{d}\le \frac{V(R)}{V(r)}\le C_{2}\biggl( \frac{R}{r}\biggr)^{D},\quad \forall 0< r< R< \infty ; \end{aligned}$$

4 $$\begin{aligned}& V(2r)\leq C_{3} V(r). \end{aligned}$$

*Throughout this paper, we always assume that* $d\ge 2$ *.*

Let $L=-\Delta +W$ be the Schrödinger operator, where Δ is the sub-Laplacian on *G* and the nonnegative potential *W* belongs to the reverse Hölder class $B_{q_{1}}$ for some $q_{1} \geq \frac{D}{2}$ and $D>3$. The Riesz transform $\mathcal{R}$ associated with the Schrödinger operator *L* is defined by

5 $$ \mathcal{R}=\nabla (-\Delta +W)^{-\frac{1}{2}}. $$

Let *b* be a locally integrable function on *G* and *T* be a linear operator. For a suitable function *f*, the commutator is defined by $[b,T]f=bT(f)-T(bf)$. Many researchers have paid attention to the commutator on $\mathbb{R}^{n}$. It is well known that Coifman, Rochberg and Weiss [4] proved that $[b,T]$ is a bounded operator on $L^{p}$ for $1< p<\infty $ if and only if $b \in \text{BMO} ( \mathbb{R}^{n})$, when *T* is a Calderón-Zygmund operator. Janson [5] proved that the commutator is bounded from $L^{p}( \mathbb{R}^{n})$ into $L^{q}(\mathbb{R}^{n})$ if and only if $b\in \operatorname{Lip}_{\nu }(\mathbb{R}^{n})$ with $\nu =(\frac{1}{p}-\frac{1}{q})n$, where $\operatorname{Lip}_{\nu }(\mathbb{R}^{n})$ is the Lipschitz space. Sheng and Liu [6] proved the boundedness of the commutator $[b,\mathcal{R}]$ from the Hardy space $H_{L}^{p}(\mathbb{R}^{n})$ into $L^{q}(\mathbb{R}^{n})$ when *b* belongs to a larger Lipschitz space. Comparatively, there has been much less research on the commutator on nilpotent Lie groups. The goal of this paper will be to obtain some estimates for the commutator related to the Schrödinger operator on nilpotent Lie groups. The complicated structure of nilpotent Lie group will bring some essential difficulties to our estimates in the following sections.

Note that a non-negative locally $L^{q}$ integrable function *W* on *G* is said to belong to $B_{q}$ ($1< q<\infty$) if there exists $C>0$ such that the reverse Hölder inequality

6 $$ \biggl( \frac{1}{\vert B \vert } \int_{B} W(y)^{q} \,\mathrm{d}y \biggr) ^{ \frac{1}{q}} \leq C \biggl( \frac{1}{\vert B \vert } \int_{B} W(y) \,\mathrm{d}y \biggr) $$

holds for every ball *B* in *G*.

We first introduce an auxiliary function as follows.

### Definition 1

Let $W\in B_{q}$ for some $q \geq \frac{D}{2}$. For $x\in G$, the function $m(x, W)$ is defined by

$$ \frac{1}{m(x, W )}=\rho (x):=\sup_{r>0}\biggl\{ r\colon \frac{r^{2}}{V(r)} \int_{B(x,r)}W(y) \,\mathrm{d}y\leq 1\biggr\} . $$

Now we define the space $\operatorname{Lip}_{\nu }^{\theta }(G)$ on the nilpotent Lie group.

### Definition 2

Let $\theta > 0$ and $0<\nu <1$, the space $\operatorname{Lip}_{\nu }^{\theta }(G)$ consists of the functions *f* satisfying

$$ \bigl\vert f(x)-f(y) \bigr\vert \leq C d^{\nu }(x,y) \biggl(1+ \frac{d(x,y)}{\rho (x)}+\frac{d(x,y)}{ \rho (y)}\biggr)^{\theta } $$

holds true for all $x,y \in G$, $x\neq y$. The norm on $\operatorname{Lip}_{\nu }^{ \theta }(G)$ is defined as follows:

$$ \Vert f \Vert _{\operatorname{Lip}_{\nu }^{\theta }(G)}\triangleq \sup_{x,y \in G, x \neq y} \biggl( \frac{\vert f(x)-f(y) \vert }{d^{\nu }(x,y)(1+\frac{d(x,y)}{ \rho (x)}+\frac{d(x,y)}{\rho (y)})^{\theta }} \biggr) < \infty . $$

It is easy to see that this space is exactly the Lipschitz space when $\theta =0$ if *G* is a stratified Lie group (cf. [7] and [8]).

We also introduce the following maximal functions.

### Definition 3

Let $f \in L^{1}_{\mathrm{loc}}(G) $. For $0 < \gamma < D$, the fractional maximal operator is defined by

$$\begin{aligned} M_{\gamma }f(x)=\sup_{x\in B}\frac{r^{\gamma }}{\vert B\vert } \int_{B}\bigl\vert f(y)\bigr\vert \,\mathrm{d}y,\quad x\in G, \end{aligned}$$

where the supremum on the right-hand side is taken over all balls $B \subseteq G$ and *r* is the radius of the ball *B*.

### Definition 4

Given $\alpha >0$, the maximal functions for $f \in L_{\mathrm{loc}}^{1}(G)$ and $x\in G$ are defined by

$$ M_{\rho ,\alpha }f(x)=\sup_{x\in B\in \mathscr{B}}\frac{1}{\vert B\vert } \int_{B}\bigl\vert f(y)\bigr\vert \,\mathrm{d}y $$

and

$$ M_{\rho ,\alpha }^{\sharp }f(x)=\sup_{x\in B\in \mathscr{B}} \frac{1}{ \vert B\vert } \int_{B}\bigl\vert f(y)-f_{B}\bigr\vert \, \mathrm{d}y, $$

where $\mathscr{B}_{\rho ,\alpha }=\{B(y,r)\colon y\in G\mbox{ and }r \leq \alpha \rho (y)\}$.

We are in a position to give the main results in this paper.

### Theorem 1

*Assume* $W \in B_{q_{1}}$ *for some* $q_{1}\geq \frac{D}{2}$, *where* *D* *denotes the dimension at infinity of the nilpotent Lie group G*. *Let*

7 $$ \frac{1}{q_{2}}= \textstyle\begin{cases} 1, & \textit{if } q_{1} \geq D, \\ 1-\frac{1}{q_{1}}+\frac{1}{D}, & \textit{if } \frac{D}{2}\leq q_{1}< D. \end{cases} $$

*Denote the adjoint operator of* $\mathcal{R}$ *by* $\tilde{\mathcal{R}}=(- \Delta +W)^{-\frac{1}{2}}\nabla $. *Then*, *for any* $h \in \operatorname{Lip}_{\nu } ^{\theta }(G)$, $0<\nu <1$, *the commutator* $[h,\tilde{\mathcal{R}}]$ *is bounded from* $L^{q}(G)$ *into* $L^{p}(G)$, $\frac{1}{p}=\frac{1}{q}-\frac{ \nu }{D}$, *if* $q_{2}< p<\infty $.

We immediately deduce Corollary 1 by duality.

### Corollary 1

*Assume* $W \in B_{q_{1}}$ *for some* $q_{1}\geq \frac{D}{2}$, *where* *D* *denotes the dimension at infinity of the nilpotent Lie group G*. *Let*

$$ \frac{1}{q_{2}}= \textstyle\begin{cases} 1, & \textit{if } q_{1} \geq D, \\ 1-\frac{1}{q_{1}}+\frac{1}{D}, & \textit{if } \frac{D}{2} \leq q_{1}< D. \end{cases} $$

*Then*, *for any* $h \in \operatorname{Lip}_{\nu }^{\theta }(G)$, $0<\nu <1$, *the commutator* $[h,\mathcal{R}]$ *is bounded from* $L^{p}(G)$ *into* $L^{q}(G)$, $\frac{1}{p}=\frac{1}{q}-\frac{\nu }{D}$, *if* $1< p< q_{2} ^{\prime}$.

Throughout this paper, unless otherwise indicated, *C* will be used to denote a positive constant that is not necessarily the same case at each occurrence and it depends at most on the constants in (3) and (6). We always denote $\delta =2-\frac{D}{q_{1}}$. By $A\sim B$, we mean that there exist constants $C>0$ and $c>0$ such that $c\leq \frac{A}{B} \leq C$.

## Estimates for the kernels of $\mathcal{R}$ and $\tilde{\mathcal{R}}$

In this section we recall some estimates for the kernels of Riesz transform $\mathcal{R}$ and the dual Riesz transform $\tilde{\mathcal{R}}$, which have been proved in [3].

### Lemma 1

$W \in B_{q}$ *is a doubling measure*, *that is*, *there exists a constant* $C>0$ *such that*

$$ \int_{B(x,2r)}W(y)\,\mathrm{d}y \leq C \int_{B(x,r)} W(y)\,\mathrm{d}y. $$

### Lemma 2

*There exists* $C>0$ *such that*, *for* $0< r< R<\infty $,

$$\begin{aligned} \frac{r^{2}}{V(r)} \int_{B(x,r)}W(y) \,\mathrm{d}y\leq C \biggl( \frac{R}{r} \biggr) ^{\frac{D}{q}-2} \frac{r^{2}}{V(R)} \int _{B(x, R)}W(y)\,\mathrm{d}y. \end{aligned}$$

### Lemma 3

*If* $r= \rho (x)$, *then*

$$\begin{aligned} \frac{r^{2}}{V(r)} \int_{B(x,r)}W(y)\,\mathrm{d}y=1. \end{aligned}$$

*Moreover*,

$$\begin{aligned} \frac{r^{2}}{V(r)} \int_{B(x, r)}W(y)\,\mathrm{d}y \sim 1 \quad \textit{if and only if}\quad r \sim \frac{1}{m(x,W)}. \end{aligned}$$

### Lemma 4

*There exist constants* $C,l_{0}>0$ *such that*

$$ \frac{1}{C} \biggl( 1+ \frac{d(x,y)}{\rho (x)} \biggr) ^{-\frac{l_{0}}{l _{0}+1}} \leq \frac{\rho (x)}{\rho (y)} \leq C \biggl( 1+ \frac{d(x,y)}{ \rho (x)} \biggr) ^{l_{0}}. $$

*In particular*, $\rho (x) \sim \rho (y)$ *if* $d(x,y)\le C\rho (x)$.

### Lemma 5

*There exist constants* $C>0$ *and* $l_{1}>0$ *such that*

$$\begin{aligned} \int_{B(x, R)}\frac{d^{2}(x,y) W(y)}{V(d(x,y)) }\,\mathrm{d}y \leq \frac{CR^{2}}{V(R)} \int_{B(x, R)}W(y)\,\mathrm{d}y \leq C \biggl( 1+ \frac{R}{\rho (x)} \biggr) ^{l_{1}}. \end{aligned}$$

Using Lemma 4, we immediately have the following lemma.

### Lemma 6

*There exist* $l_{0}>0$, $C>0$ *such that*, *for any* *x* *and* *y* *in* *G*,

$$ 1+\frac{d(x,y)}{\rho (x)}+\frac{d(x,y)}{\rho (y)} \leq C \biggl( 1+\frac{d(x,y)}{ \rho (x)} \biggr) ^{l_{0}+1}. $$

Let $\varGamma (x, y, \lambda )$ denote the fundamental solution for the operator $-\Delta +W +\lambda $, namely, $[-\Delta +W +\lambda ] \varGamma (x, y, \lambda )=\delta (y^{-1}x)$, where *δ* is the Dirac function and $\lambda \in [0,\infty )$. Markedly, $\varGamma (x, y, \lambda ) = \varGamma (y, x, \lambda )$.

### Lemma 7

*Let* *N* *be a positive integer*.

(i) *Suppose* $W\in B_{q_{1}}$ *for some* $q_{1} \geq \frac{D}{2}$. *Then there exists a constant* $C_{N} > 0$ *such that*, *for* $x \neq y$,
  $$ \bigl\vert \varGamma (x, y, \lambda )\bigr\vert \leq \frac{C_{N}}{(1+d(x,y) \lambda^{\frac{1}{2}})^{N} ( 1+d(x,y)/\rho (x) ) ^{N}} \frac{d(x,y)}{V(d(x,y))}. $$
(ii) *Suppose* $W\in B_{q_{1}}$ *for some* $q_{1}\geq \frac{D}{2}$. *Then there exists a constant* $C_{N} > 0$ *such that*
  $$\begin{aligned} \bigl\vert \nabla_{x}\varGamma (x, y, \lambda )\bigr\vert &\leq \frac{C_{N}}{(1+d(x,y) \lambda^{\frac{1}{2}})^{N} ( 1+ d(x,y) /\rho (x) ) ^{N}} \\ &\quad \times \biggl( \frac{d^{2}(x,y)}{V(d(x,y))} \int _{B(y,d(x,y) )}\frac{W(z)d(z,y)\,\mathrm{d}z}{V(d(z,y))}+ \frac{d(x,y)}{V(d(x,y))} \biggr) . \end{aligned}$$
  *Particularly*, *if* $W \in B_{q_{1}}$ *for some* $q_{1} \geq D$, *then there exists* $C_{N} > 0$ *such that*, *for* $x\neq y$,
  $$ \bigl\vert \nabla_{x}\varGamma (x, y, \lambda )\bigr\vert \leq \frac{C_{N}}{(1+d(x,y) \lambda^{\frac{1}{2}})^{N} ( 1+d(x,y) /\rho (x) ) ^{N}}\frac{d ^{2}(x,y)}{V(d(x,y))}. $$

By the functional calculus, we may write

8 $$ (-\Delta +W)^{-\frac{1}{2}}=\frac{1}{\pi } \int^{\infty }_{0} \lambda^{-\frac{1}{2}}(-\Delta +W+ \lambda )^{-1}\,\mathrm{d}\lambda . $$

Let $f \in C_{0}^{\infty }(G)$. From $(-\Delta +W+\lambda )^{-1}f(x)= \int_{G}\varGamma (x,y,\lambda )f(y)\,\mathrm{d}y$, it follows that

9 $$ \mathcal{R}f(x)= \int_{G}\mathcal{K}(x,y)f(y)\,\mathrm{d}y, $$

where

10 $$ \mathcal{K}(x,y)=\frac{1}{\pi } \int^{\infty }_{0}\lambda^{- \frac{1}{2}} \nabla_{x}\varGamma (x,y,\lambda )\,\mathrm{d}\lambda . $$

Similarly, the adjoint operator of $\mathcal{R}$ is defined to be

11 $$ \tilde{\mathcal{R}}f(x)= \int_{G}\tilde{\mathcal{K}}(x,y)f(y)\,\mathrm{d}y, $$

where

12 $$ \tilde{\mathcal{K}}(x,y)=\frac{1}{\pi }{ \int^{\infty }_{0} \lambda^{-\frac{1}{2}} \nabla_{y}\varGamma (y,x,\lambda )\,\mathrm{d} \lambda }. $$

We recall estimates of the kernels for $\mathcal{R}$ and $\tilde{\mathcal{R}}$ (cf. [3]).

### Lemma 8

*Suppose* $W \in B_{q_{1}}$ *for some* $\frac{D}{2} \leq q_{1} < D $. *For any integer* $N>0$, *there exists* $C_{N}>0$ *such that*

13 $$\begin{aligned} \bigl\vert \mathcal{K}(x,y)\bigr\vert &\leq \frac{C_{N}}{(1+ d(x,y)/\rho (x))^{N}} \biggl( \frac{d(x,y)}{V(d(x,y))} \int_{B(y,d(x,y) )}\frac{W(z)d(z,y)\,\mathrm{d}z}{V(d(z,y))} \\ &\quad{} + \frac{1}{V(d(x,y))} \biggr) \end{aligned}$$

*and*

14 $$\begin{aligned} \bigl\vert \mathcal{K}\bigl(x,y^{\prime}\bigr)-\mathcal{K}(x,y)\bigr\vert &\leq \frac{C_{N}d ^{\delta }(y,y^{\prime})}{(1+ d(x,y)/\rho (x))^{N}} \frac{d^{1-\delta }(x,y)}{V(d(x,y))} \biggl( \int_{B(y,d(x,y) )}\frac{W(z)d(z,y) \,\mathrm{d}z}{V(d(z,y))} \\ &\quad {}+\frac{1}{d(x,y)} \biggr) \end{aligned}$$

*for some* $\delta >0$ *and* $0< d(y,y^{\prime})<\frac{d(x,y)}{16}$. *If* $W \in B_{q_{1}}$ *for some* $q_{1}\geq D$, *then*

15 $$ \bigl\vert \mathcal{K}(x,y)\bigr\vert \leq \frac{C_{N}}{(1+d(x,y)/\rho (x))^{N}} \frac{1}{V(d(x,y))} $$

*and*

16 $$ \bigl\vert \mathcal{K}\bigl(x,y^{\prime}\bigr)- \mathcal{K}(x,y)\bigr\vert \leq \frac{C_{N}}{(1+ d(x,y)/\rho (x))^{N}} \frac{d^{\delta }(y,y^{\prime})}{V(d(x,y))d ^{\delta }(x,y)}. $$

### Lemma 9

*Suppose* $W \in B_{q_{1}}$ *for some* $\frac{D}{2} \leq q_{1} < D $. *For any integer* $N>0$, *there exists* $C_{N}>0$ *such that*

17 $$\begin{aligned} \bigl\vert \tilde{\mathcal{K}}(x,y)\bigr\vert &\leq \frac{C_{N}}{(1+ d(x,y)/ \rho (x))^{N}} \biggl( \frac{d(x,y)}{V(d(x,y))}\int_{B(y,d(x,y))}\frac{W(z)d(z,y) \,\mathrm{d}z}{V(d(z,y))} \\ &\quad {}+\frac{1}{V(d(x,y))} \biggr) \end{aligned}$$

*and*

18 $$\begin{aligned} \bigl\vert \tilde{\mathcal{K}}\bigl(x^{\prime},y\bigr)- \tilde{\mathcal{K}}(x,y)\bigr\vert &\leq \frac{C_{N}d^{\delta }(x,x^{\prime})}{(1+ d(x,y)/\rho (x))^{N}} \frac{d ^{1-\delta }(x,y)}{V(d(x,y))} \biggl( \int_{B(y,d(x,y) )}\frac{W(z)d(z,y) \,\mathrm{d}z}{V(d(z,y))} \\ &\quad {}+\frac{1}{d(x,y)} \biggr) \end{aligned}$$

*for some* $\delta >0$ *and* $0< d(x,x^{\prime})<\frac{d(x,y)}{16}$. *If* $W \in B_{q_{1}}$ *for some* $q_{1}\geq D$, *then*

19 $$ \bigl\vert \tilde{\mathcal{K}}(x,y)\bigr\vert \leq \frac{C_{N}}{(1+d(x,y)/ \rho (x))^{N}}\frac{1}{V(d(x,y))} $$

*and*

20 $$ \bigl\vert \tilde{\mathcal{K}}\bigl(x^{\prime},y\bigr)- \tilde{\mathcal{K}}(x,y)\bigr\vert \leq \frac{C_{N}}{(1+ d(x,y)/\rho (x))^{N}} \frac{d^{\delta }(x,x^{\prime})}{V(d(x,y))d ^{\delta }(x,y)}. $$

## Some technical lemmas and propositions

### Proposition 1

*Let* $\theta > 0$ *and* $1 \leq s <\infty $. *If* $h \in \operatorname{Lip}_{\nu }^{ \theta }(G)$, *then there exists a positive constant* *C* *such that*, *for all* $B = B(x, r)$ *with* $x\in G$ *and* $r > 0$,

$$ \biggl( \frac{1}{\vert B\vert } \int_{B}\bigl\vert h(y)-h_{B}\bigr\vert ^{s} \,\mathrm{d}y \biggr) ^{1/s}\leq C\Vert h\Vert _{\operatorname{Lip} _{\nu } ^{\theta }(G)}r^{\nu } \biggl( 1+\frac{r}{\rho (x)} \biggr) ^{(l_{0}+1)\theta }. $$

### Proof

Since $h \in \operatorname{Lip}_{\nu }^{\theta }(G)$, then

$$\begin{aligned} & \biggl( \frac{1}{\vert B(x,r)\vert } \int_{ B(x,r)}\bigl\vert h(y)-h _{B}\bigr\vert ^{s} \,\mathrm{d}y \biggr) ^{\frac{1}{s}} \\ &\quad \leq \biggl( \frac{1}{\vert B(x,r)\vert } \int_{ B(x,r)} \bigl\vert h(y)-h(x)\bigr\vert ^{s} \,\mathrm{d}y \biggr) ^{\frac{1}{s}}+ \biggl( \frac{1}{ \vert B(x,r)\vert } \int_{ B(x,r)}\bigl\vert h(x)-h_{B}\bigr\vert ^{s} \,\mathrm{d}y \biggr) ^{\frac{1}{s}} \\ &\quad \leq 2C \biggl( \frac{1}{\vert B(x,r)\vert } \int_{ B(x,r)} \biggl\vert \Vert h\Vert _{\operatorname{Lip} _{\nu }^{\theta }(G)}d^{\nu }(x,y) \biggl( 1+\frac{d(x,y)}{ \rho (x)}+\frac{d(x,y)}{\rho (y)} \biggr) ^{\theta }\biggr\vert ^{s} \, \mathrm{d}y \biggr) ^{\frac{1}{s}} \\ &\quad \leq C\Vert h\Vert _{\operatorname{Lip}_{\nu }^{\theta }(G)}r^{\nu } \biggl( 1+ \frac{r}{ \rho (x)}+\frac{r}{\rho (y)} \biggr) ^{\theta } \\ &\quad \leq C\Vert h\Vert _{\operatorname{Lip} _{\nu }^{\theta }(G)}r^{\nu } \biggl( 1+ \frac{r}{ \rho (x)} \biggr) ^{ ( l_{0}+1 ) \theta }, \end{aligned}$$

where we have used Lemma 6 in the penultimate inequality. □

Similar to the proof of Proposition 1, we immediately get the following.

### Lemma 10

*Let* $h \in \operatorname{Lip}_{\nu }^{\theta }(G)$, $B = B(x, r)$ *and* $s \geq 1$. *Then there exists a positive constant* *C* *such that*, *for all* $k\in N$,

$$ \biggl( \frac{1}{\vert 2^{k}B\vert } \int_{2^{k}B}\bigl\vert h(y)-h _{B}\bigr\vert ^{s} \,\mathrm{d}y \biggr) ^{\frac{1}{s}}\leq C\Vert h\Vert _{\operatorname{Lip}_{\nu }^{\theta }(G)}2^{k\nu }r^{\nu } \biggl( 1+\frac{2^{k}r}{ \rho (x)} \biggr) ^{ ( l_{0}+1 ) \theta }. $$

### Proposition 2

*Let* $W\in B_{q_{1}}$ *for* $q_{1} \geq \frac{D}{2}$. *Let*

$$ \frac{1}{q_{2}}= \textstyle\begin{cases} 1, & \textit{if } q_{1}\geq D, \\ 1 - \frac{1}{q_{1}}+\frac{1}{D}, & \textit{if } \frac{D}{2}\leq q_{1}< D. \end{cases} $$

*Then there exists* $C>0$ *such that*, *for any* $f \in C_{0}^{\infty }(G)$,

$$ \bigl\Vert \mathcal{R}f(x)\bigr\Vert _{L^{p}(G)} \leq C \Vert f \Vert _{L^{p}(G)}, $$

*where* $1< p< q'_{2}$.

### Proposition 3

*Let* $W\in B_{q_{1}}$ *for* $q_{1} \geq \frac{D}{2}$. *Let*

$$ \frac{1}{q_{2}}= \textstyle\begin{cases} 1,& \textit{if } q_{1}\geq D, \\ 1 - \frac{1}{q_{1}}+\frac{1}{D},&\textit{if } \frac{D}{2}\leq q_{1}< D. \end{cases} $$

*Then there exists* $C>0$ *such that*, *for any* $f \in C_{0}^{\infty }(G)$,

$$ \bigl\Vert \tilde{\mathcal{R}}f(x)\bigr\Vert _{L^{p}(G)} \leq C \Vert f\Vert _{L^{p}(G)}, $$

*where* $q_{2}< p<\infty $.

For the proofs of Proposition 2 and Proposition 3, one can refer to [3].

### Proposition 4

*There exists a sequence of points* $\{x_{k}\}_{k=1}^{\infty }$ *in* *G*, *so that the set of critical balls* $Q_{k} = B(x_{k}, \rho (x_{k}))$, $k \geq 1$, *satisfies*

(i) $\bigcup_{k} Q_{k} = G$;
(ii) *There exists* *N* *such that*, *for every* $k\in N$, $\sharp \{j\colon 4Q_{j}\cap 4Q_{k}\neq \varnothing \}\leq N$.

### Proposition 5

*For* $1 < p < \infty $, *there exist positive constants* *C*, *α* *and* *β* *such that if* $\{Q_{k}\}_{k=1}^{\infty }$ *is a sequence of balls as in Proposition* 4, *then*

$$ \int_{G}\bigl\vert M_{\rho ,\alpha }f(x)\bigr\vert ^{p}\,\mathrm{d}x \leq C \int_{G}\bigl\vert M_{\rho ,\beta }^{\sharp }f(x) \bigr\vert ^{p}\,\mathrm{d}x+C\sum_{k} \vert Q_{k}\vert \biggl( \frac{1}{\vert Q _{k}\vert } \int_{2Q_{k}}\bigl\vert f(x)\bigr\vert \,\mathrm{d}x \biggr) ^{p} $$

*for all* $f\in L_{\mathrm{loc}}^{1}(G)$.

The above propositions have been proved in [9] and [10] in the case of a homogeneous space, respectively.

### Lemma 11

*Let* $W\in B_{q_{1}}$ *for* $q_{1} \geq \frac{D}{2}$, *and let*

$$ \frac{1}{q_{2}}= \textstyle\begin{cases} 1,& \textit{if }q_{1}\geq D, \\ 1 - \frac{1}{q_{1}}+\frac{1}{D}, & \textit{if }\frac{D}{2}\leq q_{1}< D \end{cases} $$

*and* $h\in \operatorname{Lip}_{\nu }^{\theta }(G)$. *Then*, *for* $q_{2}< m<\infty $, *there exists a positive constant* *C* *such that*

$$ \frac{1}{\vert Q\vert } \int_{Q}\bigl\vert [h, \tilde{\mathcal{R}}]f(x)\bigr\vert \,\mathrm{d}x\leq C\Vert h \Vert _{\operatorname{Lip}_{\nu } ^{\theta }(G)}\inf_{y\in Q} \bigl\{ M_{m\nu }\bigl(\vert f\vert ^{m}\bigr) (y)\bigr\} ^{ \frac{1}{m}} $$

*holds true for all* $f\in L_{\mathrm{loc}}^{m}(G)$ *and every ball* $Q=B(x_{0}, \rho (x_{0}))$, *where* $M_{m\nu }$ *is a fractional maximal operator*.

### Proof

Throughout the proof of the lemma, we always assume $\frac{D}{2}\leq q_{1}< D$. Let $f\in L^{p}(G)$ and $Q=B(x_{0},\rho (x _{0}))$. For

21 $$ [h,\tilde{\mathcal{R}}]f=(h-h_{Q})\tilde{ \mathcal{R}}f- \tilde{\mathcal{R}}\bigl((h-h_{Q})f\bigr), $$

we need to consider the average on *Q* for each term. By the Hölder inequality with $m>q_{2}$ and Proposition 1,

$$\begin{aligned} \frac{1}{\vert Q\vert } \int_{Q}\bigl\vert (h-h_{Q}) \tilde{ \mathcal{R}}f(x)\bigr\vert \,\mathrm{d}x &\leq \biggl( \frac{1}{ \vert Q\vert } \int_{Q}\bigl\vert \bigl( h(x)-h_{Q} \bigr) \bigr\vert ^{m^{\prime}} \,\mathrm{d}x \biggr) ^{1/m^{\prime}} \biggl( \frac{1}{\vert Q\vert } \int_{Q}\bigl\vert \tilde{\mathcal{R}}f(x)\bigr\vert ^{m}\, \mathrm{d}x \biggr) ^{1/m} \\ &\leq C\Vert h\Vert _{\operatorname{Lip}_{\nu }^{\theta }(G)} \bigl( \rho (x_{0}) \bigr) ^{ \nu } \biggl( \frac{1}{\vert Q\vert } \int_{Q}\bigl\vert \tilde{\mathcal{R}}f(x)\bigr\vert ^{m}\,\mathrm{d}x \biggr) ^{1/m}. \end{aligned}$$

If we write $f = f_{1} + f_{2}$ with $f_{1} = f_{\chi_{2Q}}$, due to Proposition 3, we get

22 $$\begin{aligned} \bigl(\rho (x_{0})\bigr)^{\nu } \biggl( \frac{1}{\vert Q\vert } \int_{Q} \bigl\vert \tilde{\mathcal{R}}f_{1}(x) \bigr\vert ^{m}\,\mathrm{d}x \biggr) ^{1/m} &\leq C\bigl( \rho (x_{0})\bigr)^{\nu } \biggl( \frac{1}{\vert 2Q\vert } \int_{2Q}\bigl\vert f(x)\bigr\vert ^{m}\, \mathrm{d}x \biggr) ^{1/m} \\ & \leq C \inf_{y\in Q} \bigl\{ M_{m\nu }\bigl(\vert f \vert ^{m}\bigr) (y) \bigr\} ^{\frac{1}{m}} \end{aligned}$$

for $x\in Q$, and using (17) in Lemma 9, we split $\tilde{\mathcal{R}}f_{2}(x)$ into two parts

$$\begin{aligned} \bigl\vert \tilde{\mathcal{R}}f_{2}(x)\bigr\vert &=\biggl\vert \int _{d(x_{0},z) >2\rho (x_{0})}\tilde{\mathcal{\mathcal{K}}}(x,z)f(z) \,\mathrm{d}z \biggr\vert \leq C\bigl\{ I_{1}(x)+I_{2}(x)\bigr\} , \end{aligned}$$

where

$$ I_{1}(x)= \int_{ d(x_{0},z) >2\rho (x_{0})}\frac{\vert f(z)\vert }{V(d(x,z))(1+d(x,z) / \rho (x))^{N}} \,\mathrm{d}z $$

and

$$ I_{2}(x)= \int_{ d(x_{0},z) >2\rho (x_{0})}\frac{d(x,z)\vert f(z)\vert }{V(d(x,z))(1+d(x,z) / \rho (x))^{N}} \int _{B(z,\frac{d(x,z)}{4})}\frac{d(u,z)W(u)}{V(d(u,z))} \, \mathrm{d}u \,\mathrm{d}z. $$

To deal with $I_{1}(x)$, noting that $\rho (x) \sim \rho (x_{0})$ and $d(z,x) \sim d(z,x_{0})$, we split $d(z,x_{0})>2 \rho (x_{0})$ into annuli to obtain

23 $$\begin{aligned} \bigl( \rho (x_{0}) \bigr) ^{\nu }I_{1}(x) &= \bigl( \rho (x_{0}) \bigr) ^{\nu } \int_{ d(x_{0},z) >2\rho (x_{0})}\frac{\vert f(z)\vert }{V(d(x,z))(1+d(x,z) / \rho (x))^{N}} \,\mathrm{d}z \\ & \leq C \sum_{k \geq 1}\frac{2^{-Nk} ( \rho (x_{0}) ) ^{ \nu }}{V(2^{k}\rho (x_{0}))} \int_{d(x_{0},z) < 2^{k}\rho (x _{0})} \bigl\vert f(z)\bigr\vert \,\mathrm{d}z \\ & \leq C \inf_{y\in Q} \bigl\{ M_{m\nu } \bigl( \vert f \vert ^{m} \bigr) ( y ) \bigr\} ^{\frac{1}{m}}. \end{aligned}$$

Secondly, we consider the term $I_{2}(x)$. We have, for $x \in Q$,

$$\begin{aligned} \bigl( \rho (x_{0}) \bigr) ^{\nu }I_{2}(x) &= \bigl( \rho (x_{0}) \bigr) ^{\nu } \int_{ d(x_{0},z) >2\rho (x_{0})}\frac{d(x,z)\vert f(z)\vert }{V(d(x,z))(1+d(x,z) / \rho (x))^{N}} \\ &\quad {}\times\int _{B(z,\frac{d(x,z)}{4})}\frac{d(u,z)W(u)}{V(d(u,z))} \, \mathrm{d}u \,\mathrm{d}z \\ & \leq C \bigl( \rho (x_{0}) \bigr) ^{\nu } \int _{ d(x_{0},z) >2\rho (x_{0})}\frac{d(x,z)\vert f(z)\vert }{V(d(x,z))(1+d(x,z) / \rho (x))^{N}} \\ &\quad {}\times \int_{B(z,4d(x_{0},z))} \frac{d(u,z)W(u)}{V(d(u,z))} \,\mathrm{d}u \,\mathrm{d}z \\ & \leq C \bigl( \rho (x_{0}) \bigr) ^{\nu } \sum _{k \geq 1}\frac{2^{-Nk}2^{k} \rho (x_{0})}{V(2^{k}\rho (x_{0}))} \int _{d(x_{0},z)< 2^{k+1}\rho (x_{0})} \bigl\vert f(z)\bigr\vert \\ &\quad {}\times \int _{B(z,2^{k+3}d(x_{0},z))}\frac{d(u,z)W(u)}{V(d(u,z))} \, \mathrm{d}u \,\mathrm{d}z \\ & \leq C \bigl( \rho (x_{0}) \bigr) ^{\nu } \sum _{k \geq 1}\frac{2^{-Nk}2^{k} \rho (x_{0})}{V(2^{k}\rho (x_{0}))} \\ &\quad {}\times \int _{d(x_{0},z)< 2^{k+1}\rho (x_{0})} \bigl\vert f(z)\bigr\vert \mathcal{I}_{1}(W_{\chi_{B(x_{0},2^{k}\rho (x_{0}))}}) (z) \, \mathrm{d} z. \end{aligned}$$

Let $q_{2}< m< D$. Using the Hölder inequality and the boundedness of the fractional integral $\mathcal{I}_{1} \colon L^{m'} \mapsto L^{q _{1}}$ with

$$ \frac{1}{q_{1}}= \frac{1}{m^{\prime}}-\frac{1}{\beta }, $$

where

$$ \beta = \textstyle\begin{cases} d, & \textit{if } 2^{k}\rho (x_{0})< 1, \\ D, & \textit{if } 2^{k}\rho (x_{0}) \geq 1 \end{cases} $$

(cf. Theorem 1.6 in [11]), we obtain

$$\begin{aligned} &\int_{d(x_{0},z)< 2^{k+1}\rho (x_{0})} \bigl\vert f(z)\bigr\vert \mathcal{I}_{1}(W \chi_{B(x_{0},2^{k}\rho (x_{0}))}) (z) \,\mathrm{d}z \\ &\quad \leq \Vert f\chi_{B(x_{0},2^{k}\rho (x_{0}))}\Vert _{m}\bigl\Vert \mathcal{I} _{1}(W\chi_{B(x_{0},2^{k}\rho (x_{0}))}) \bigr\Vert _{m^{\prime}} \\ &\quad \leq \Vert f\chi_{B(x_{0},2^{k}\rho (x_{0}))}\Vert _{m}\Vert W \chi_{B(x_{0},2^{k}\rho (x_{0}))}\Vert _{q_{1}}. \end{aligned}$$

Since $W\in B_{q_{1}}$, we obtain

$$\begin{aligned} \Vert W\chi_{B(x_{0},2^{k}\rho (x_{0}))}\Vert _{q_{1}} &\leq C \bigl(V \bigl(2^{k}\rho (x_{0})\bigr) \bigr)^{-\frac{1}{q_{1}^{\prime}}} \int_{B(x_{0},2^{k}\rho (x_{0}))} W \\ &\leq C\bigl(2^{k}\rho (x_{0})\bigr)^{-2} \bigl(V\bigl(2^{k}\rho (x_{0})\bigr) \bigr)^{1-\frac{1}{q _{1}^{\prime}}} \frac{(2^{k}\rho (x_{0}))^{2}}{V(2^{k}\rho (x_{0}))} \int_{B(x_{0},2^{k}\rho (x_{0}))} W \\ &\leq C\bigl(2^{k}\bigr)^{l_{1}-2} \bigl(V\bigl(2^{k} \rho (x_{0})\bigr) \bigr)^{1-\frac{1}{q _{1}^{\prime}}}\bigl(\rho (x_{0}) \bigr)^{-2}, \end{aligned}$$

where in the last two inequalities we have used doubling measure and the definition of *ρ*, respectively. Therefore,

$$ I_{2}(x)\leq V\bigl(\rho (x_{0})\bigr)^{-1/q_{1}^{\prime}-1}\sum _{k\geq 1}2^{k(-N+l _{1}-1-d/q_{1}^{\prime})}\Vert f\chi_{B(x_{0},2^{k}\rho (x_{0}))} \Vert _{m}. $$

Finally, observing that

$$ \Vert f\chi_{B(x_{0},2^{k}\rho (x_{0}))}\Vert _{m}\leq \bigl(V \bigl(2^{k} \rho (x_{0})\bigr)\bigr)^{\frac{1}{m}}\inf _{y\in Q}M_{m}f(y) $$

and using that $\frac{1}{q_{1}^{\prime}}-\frac{1}{m}= \frac{1}{D}$ or $\frac{1}{d}$, we have

$$ \bigl(\rho (x_{0})\bigr)^{\nu }I_{2}(x)\leq C\inf _{y\in Q}\bigl\{ M_{m\nu }\bigl(\vert f\vert ^{m}\bigr) (y)\bigr\} ^{\frac{1}{m}} $$

by choosing *N* large enough.

So far, we have solved the term $(h-h_{Q})\mathcal{R}f$, now we want to control $\int \mathcal{R}[(h-h_{Q})f]\,\mathrm{d}x$ by the term $C \inf_{y\in Q}\{M_{m\nu }(\vert f \vert ^{m})(y)\}^{\frac{1}{m}}$. We still split $f=f_{1}+f_{2}$. Choose $q_{2}<\tilde{m}<m$ and set $t=\frac{\tilde{m}m}{m-\tilde{m}}$. Using the boundedness of $\tilde{\mathcal{R}}$ on $L^{\tilde{m}}(G)$ and the Hölder inequality, we get

$$\begin{aligned} &\frac{1}{\vert Q\vert } \int_{Q}\bigl\vert \tilde{\mathcal{R}}(h-h _{Q})f_{1}(x)\bigr\vert \,\mathrm{d}x \\ &\quad \leq \biggl( \frac{1}{\vert Q\vert } \int_{Q}\bigl\vert \tilde{\mathcal{R}}(h-h_{Q})f_{1}(x) \bigr\vert ^{\tilde{m}} \,\mathrm{d}x \biggr) ^{1/\tilde{m}} \\ & \quad \leq C \biggl( \frac{1}{\vert Q\vert } \int_{Q}\bigl\vert (h-h _{Q})f(x)\bigr\vert ^{\tilde{m}} \,\mathrm{d}x \biggr) ^{1/\tilde{m}} \\ &\quad \leq C \biggl( \frac{1}{\vert Q \vert } \int_{2Q}\bigl\vert f(x)\bigr\vert ^{m} \, \mathrm{d}x \biggr) ^{1/m} \biggl( \frac{1}{\vert Q \vert } \int_{2Q}\bigl\vert h(x)-h _{Q}\bigr\vert ^{t}\,\mathrm{d}x \biggr) ^{1/t} \\ &\quad \leq C \biggl( \frac{(\rho (x_{0}))^{m\nu }}{\vert Q \vert } \int_{2Q} \bigl\vert f(x)\bigr\vert ^{m} \, \mathrm{d}x \biggr) ^{1/m} \Vert h\Vert _{\operatorname{Lip} _{\nu }^{\theta }(G)} \biggl( 1+ \frac{\rho (x_{0})}{\rho (x_{0})} \biggr) ^{(l_{0}+1)\theta } \\ & \quad \leq C \Vert h\Vert _{\operatorname{Lip}_{\nu }^{\theta }(G)}\inf_{y\in Q} \bigl\{ M_{m\nu } \bigl( \vert f \vert ^{m} \bigr) ( y ) \bigr\} ^{ \frac{1}{m}}, \end{aligned}$$

where we have applied Proposition 1 to the last but one inequality. Similarly, for $x\in Q$ and using (17) in Lemma 9, we have

$$\begin{aligned} \bigl\vert \tilde{R}(h-h_{Q})f_{2}(x) \bigr\vert &= \biggl\vert \int _{d(x_{0},z)>2\rho (x_{0})} \tilde{\mathcal{\mathcal{K}}}(x,z) \bigl(h(z)-h _{Q}\bigr)f(z)\,\mathrm{d}z\biggr\vert \leq C \bigl\{ \tilde{I}_{1}(x)+\tilde{I}_{2}(x) \bigr\} , \end{aligned}$$

where

$$ \tilde{I}_{1}(x)= \int_{ d(x_{0},z) >2\rho (x_{0})}\frac{\vert (h-h_{Q})f(z)\vert }{V(d(x,z))(1+d(x,z) / \rho (x))^{N}} \, \mathrm{d}z $$

and

$$ \tilde{I}_{2}(x)= \int_{ d(x_{0},z) >2\rho (x_{0})}\frac{\vert (h-h_{Q})f(z)\vert }{V(d(x,z))(1+d(x,z) / \rho (x))^{N}} \int _{B(z,\frac{d(x,z)}{4})}\frac{d(u,z)W(u)}{V(d(u,z))} \, \mathrm{d}u \,\mathrm{d}z. $$

We start by observing that for $1\leq \tilde{m}< m$, $t= \frac{\tilde{m}m}{m- \tilde{m}}$, and by Lemma 6,

24 $$\begin{aligned} &\bigl\Vert \bigl[(h-h_{Q})f\bigr] { \chi_{B(x_{0},2^{k}\rho (x_{0}))}}\bigr\Vert _{ \tilde{m}} \\ & \quad \leq \Vert f_{\chi_{B(x_{0},2^{k}\rho (x_{0}))}}\Vert _{m}\bigl\Vert (h-h _{Q}){\chi_{B(x_{0},2^{k}\rho (x_{0}))}}\bigr\Vert _{t} \\ & \quad \leq C \biggl( \int_{B(x_{0},2^{k}\rho (x_{0}))}\bigl\vert f(y)\bigr\vert ^{m}\, \mathrm{d}y \biggr) ^{1/m} \Vert h\Vert _{\operatorname{Lip}_{\nu }^{\theta }(G)} \bigl(2^{k}\rho (x_{0})\bigr)^{ \nu } \bigl(1+2^{k}\bigr)^{(l_{0}+1)\theta }V^{1/t}\bigl(2^{k} \rho (x_{0})\bigr) \\ & \quad \leq C 2^{k(l_{0}+1)\theta }V^{1/\tilde{m}}\bigl(2^{k}\rho (x_{0})\bigr)\Vert h \Vert _{\operatorname{Lip} _{\nu }^{\theta }(G)}\inf _{y\in Q} \bigl\{ M_{m\nu } \bigl( \vert f \vert ^{m} \bigr) ( y ) \bigr\} ^{\frac{1}{m}}. \end{aligned}$$

For $\tilde{I}_{1}(x)$, using (24) with $\tilde{m}=1$, we have

$$\begin{aligned} \tilde{I}_{1}(x) & \leq C\sum_{k \geq 1} \frac{2^{-Nk}}{V(2^{k}\rho (x _{0}))} \int_{d(x_{0},z)>2^{k}\rho (x_{0})}\bigl\vert h(z)-h_{Q}\bigr\vert \bigl\vert f(z) \bigr\vert \,\mathrm{d}z \\ & \leq C \Vert h \Vert _{\operatorname{Lip}_{\nu }^{\theta }(G)}\inf_{y\in Q} \bigl\{ M_{m \nu } \bigl( \vert f \vert ^{m} \bigr) ( y ) \bigr\} ^{\frac{1}{m}} \sum_{k \geq 1}2^{k(-N+(l_{0}+1)\theta )} \\ & \leq C \Vert h \Vert _{\operatorname{Lip}_{\nu }^{\theta }(G)}\inf_{y\in Q} \bigl\{ M_{m \nu } \bigl( \vert f \vert ^{m} \bigr) ( y ) \bigr\} ^{\frac{1}{m}} \end{aligned}$$

if we choose *N* sufficiently large.

To deal with $\tilde{I}_{2}(x)$, we discuss as in the estimate for $I_{2}(x)$ with $(h-h_{Q})f$ instead of *f* and *m̃* and $\tilde{q_{1}}$ instead of *m* and $q_{1}$, but we cannot avoid to discuss the different cases where $2^{k}\rho (x_{0}) \geq 1$ and $2^{k}\rho (x_{0})< 1$. Let

$$ \frac{1}{\tilde{m}}= \textstyle\begin{cases} \frac{1}{\tilde{q_{1}}}-\frac{1}{d}, & \textit{if } 2^{k}\rho (x_{0})< 1, \\ \frac{1}{\tilde{q_{1}}}-\frac{1}{D}, & \textit{if } 2^{k}\rho (x_{0}) \geq 1. \end{cases} $$

Using (24), we similarly have

$$\begin{aligned} \tilde{I}_{2}(x) \leq C \Vert h \Vert _{\operatorname{Lip}_{\nu }^{\theta }(G)}\inf _{y\in Q} \bigl\{ M_{m\nu } \bigl( \vert f \vert ^{m} \bigr) ( y ) \bigr\} ^{ \frac{1}{m}}, \end{aligned}$$

where we choose *N* large enough to ensure the above series converges. □

### Lemma 12

*Let* $\tilde{\mathcal{R}}=(-\Delta +W)^{-\frac{1}{2}}\nabla $ *be the adjoint operator of the Riesz transform* $\mathcal{R}$. *Then there exists* $C>0$ *such that*, *for any* $f\in L_{\mathrm{loc}}^{m}(G)$ *and* $h \in \operatorname{Lip}_{\nu } ^{\theta }(G)$,

25 $$ M_{\rho ,\alpha }^{\sharp }\bigl([h,\tilde{R}]f\bigr) (x) \leq C \Vert h \Vert _{\operatorname{Lip}_{ \nu }^{\theta }(G)}\bigl( \bigl\{ M_{m\nu } \bigl( \vert f \vert ^{m} \bigr) ( y ) \bigr\} ^{\frac{1}{m}}+\bigl\{ M_{m\nu}\bigl(| \tilde{\mathcal{R}}f| ^{m}\bigr)(x)\bigr\} ^{\frac{1}{m}}\bigr). $$

### Proof

Let $f\in L_{\mathrm{loc}}^{m}(G)$, $x\in G$ and a ball $B=B(x_{0},r)$ with $x\in B$ and $r<\epsilon \rho (x_{0})$, $\epsilon >0$, we need to control $J=\frac{1}{\vert B \vert }\int_{B}\vert [h,\tilde{\mathcal{R}}]f(y)-c \vert \,\mathrm{d}y$ by the right-hand side of (25) for some constant *c*, which will be designated later. Let $f=f_{1}+f_{2}$, where $f_{1}=f_{\chi_{2B}}$ and $f_{2}=f-f_{1}$. Then

$$\begin{aligned}{} [h,\tilde{\mathcal{R}}]f &=[h-h_{2B},\tilde{\mathcal{R}}]f \\ &=(h-h_{2B}) \tilde{\mathcal{R}}f-\tilde{\mathcal{R}}(h-h_{2B})f_{1}- \tilde{\mathcal{R}}(h-h_{2B})f_{2} \\ & \doteq A_{1}f+A_{2}f+A_{3}f. \end{aligned}$$

Take $c=\int_{d(x_{0},z)\geq 2r}\tilde{\mathcal{K}}(x_{0},z)(h(z)-h _{2B})f(z)\,\mathrm{d}z$. Then we have

$$\begin{aligned} J & \leq \frac{1}{\vert B \vert } \int_{B}\bigl\vert A_{1}f(y)\bigr\vert \, \mathrm{d}y + \frac{1}{\vert B \vert } \int_{B}\bigl\vert A_{2}f(y)\bigr\vert \, \mathrm{d}y+ \frac{1}{\vert B \vert } \int_{B}\bigl\vert A_{3}f(y)-c\bigr\vert \, \mathrm{d}y \\ & \doteq J_{1}+J_{2}+J_{3}. \end{aligned}$$

At first, we consider $J_{1}$. Note that $d(x,x_{0})\leq r<\epsilon \rho (x_{0})$ implies $\rho (x)\sim \rho (x_{0})$. By the Hölder inequality, Proposition 1 and Proposition 3, we have

$$\begin{aligned} J_{1} &= \frac{1}{\vert B \vert } \int_{B}\bigl\vert A_{1}f(y)\bigr\vert \, \mathrm{d}y \\ &= \frac{1}{\vert B \vert } \int_{B}\bigl\vert \bigl(h(y)-h_{2B}\bigr) \tilde{\mathcal{R}}f(y)\bigr\vert \,\mathrm{d}y \\ &\leq \frac{C}{\vert B \vert } \biggl[ \int_{B}\bigl\vert h(y)-h_{2B} \bigr\vert ^{\frac{m}{m-1}} \,\mathrm{d}y \biggr] ^{\frac{m-1}{m}} \biggl[ \int_{B}\bigl\vert \tilde{\mathcal{R}}f(y) \bigr\vert ^{m}\,\mathrm{d}y \biggr] ^{\frac{1}{m}} \\ &\leq \frac{C}{\vert B \vert } \biggl[ \int_{2B}\bigl\vert h(y)-h_{2B} \bigr\vert ^{ \frac{m}{m-1}}\,\mathrm{d}y \biggr] ^{\frac{m-1}{m}} \biggl[ \int _{B}\bigl\vert \tilde{\mathcal{R}}f(y) \bigr\vert ^{m}\,\mathrm{d}y \biggr] ^{\frac{1}{m}} \\ &\leq \frac{C}{\vert B \vert }\Vert h\Vert _{\operatorname{Lip}_{\nu }^{\theta }(G)} \biggl( 1+ \frac{2r}{ \rho (x_{0})} \biggr) ^{(l_{0}+1)\theta } r^{\nu }\vert B\vert ^{ \frac{m-1}{m}} \biggl[ \int_{B}\bigl\vert \tilde{\mathcal{R}}f(y) \bigr\vert ^{m}\, \mathrm{d}y \biggr] ^{\frac{1}{m}} \\ &\leq \frac{C}{\vert B \vert }\Vert h\Vert _{\operatorname{Lip}_{\nu }^{\theta }(G)} \biggl( 1+ \frac{2 \epsilon \rho (x_{0})}{\rho (x_{0})} \biggr) ^{(l_{0}+1)\theta } r^{ \nu }\vert B\vert ^{\frac{m-1}{m}} \biggl[ \int_{B}\bigl\vert \tilde{\mathcal{R}}f(y) \bigr\vert ^{m}\,\mathrm{d}y \biggr] ^{\frac{1}{m}} \\ &\leq C^{\prime} \Vert h\Vert _{\operatorname{Lip}_{\nu }^{\theta }(G)} \biggl( \frac{r ^{m\nu }}{\vert B \vert } \int_{B}\bigl\vert \tilde{\mathcal{R}} f(y) \bigr\vert ^{m} \, \mathrm{d}y \biggr) ^{ \frac{1}{m}} \\ & \leq C^{\prime} \Vert h\Vert _{\operatorname{Lip}_{\nu }^{\theta }(G)} \bigl\{ M_{m \nu } \bigl( \vert \tilde{\mathcal{R}} f \vert ^{m} \bigr) ( x ) \bigr\} ^{\frac{1}{m}} \end{aligned}$$

for $m>q_{2}$.

For $J_{2}$, by the Hölder inequality and Proposition 3,

$$\begin{aligned} J_{2} &\leq \frac{1}{\vert B \vert } \int_{B} \bigl\vert A_{2}f(y) \bigr\vert \, \mathrm{d}y \\ & \leq \biggl( \frac{1}{\vert B \vert } \int_{B} \bigl\vert A_{2}f(y) \bigr\vert ^{\tilde{m}}\, \mathrm{d}y \biggr) ^{\frac{1}{\tilde{m}}} \\ & \leq C \biggl( \frac{1}{\vert B \vert } \biggr) ^{\frac{1}{\tilde{m}}} \biggl( \int_{2B}\bigl\vert \bigl(h(y)-h_{2B}\bigr)f(y) \bigr\vert ^{\tilde{m}}\, \mathrm{d}y \biggr) ^{\frac{1}{\tilde{m}}} \\ & \leq \frac{C}{\vert B \vert ^{\frac{1}{\tilde{m}}}} \biggl( \int_{2B} \bigl\vert h(y)-h_{2B}\bigr\vert ^{\frac{m\tilde{m}}{m-\tilde{m}}} \, \mathrm{d}y \biggr) ^{\frac{1}{\tilde{m}}-\frac{1}{m}} \biggl( \int _{2B}\bigl\vert f(y) \bigr\vert ^{m}\, \mathrm{d}y \biggr) ^{\frac{1}{m}} \\ & \leq C \Vert h\Vert _{\operatorname{Lip}_{\nu }^{\theta }(G)}r^{\nu } \biggl( 1+ \frac{2r}{ \rho (x_{0})} \biggr) ^{(l_{0}+1)\theta } \biggl( \frac{1}{\vert B \vert } \biggr) ^{\frac{1}{m}} \biggl( \int_{2B}\bigl\vert f(y) \bigr\vert ^{m}\, \mathrm{d}y \biggr) ^{\frac{1}{m}} \\ & \leq C \Vert h\Vert _{\operatorname{Lip}_{\nu }^{\theta }(G)}r^{\nu } \biggl( 1+ \frac{2 \epsilon \rho (x_{0})}{\rho (x_{0})} \biggr) ^{(l_{0}+1)\theta } \biggl( \frac{1}{\vert B \vert } \biggr) ^{\frac{1}{m}} \biggl( \int_{2B}\bigl\vert f(y) \bigr\vert ^{m}\, \mathrm{d}y \biggr) ^{\frac{1}{m}} \\ & \leq C^{\prime} \Vert h\Vert _{\operatorname{Lip}_{\nu }^{\theta }(G)} \biggl( \frac{r ^{m\nu }}{\vert B \vert } \int_{2B}\bigl\vert f(y) \bigr\vert ^{m}\, \mathrm{d}y \biggr) ^{ \frac{1}{m}} \\ & \leq C^{\prime} \Vert h\Vert _{\operatorname{Lip}_{\nu }^{\theta }(G)} \biggl( \frac{(2r)^{m \nu }}{\vert 2B \vert } \int_{2B}\bigl\vert f(y) \bigr\vert ^{m}\, \mathrm{d}y \biggr) ^{ \frac{1}{m}} \\ & \leq C^{\prime} \Vert h\Vert _{\operatorname{Lip}_{\nu }^{\theta }(G)} \bigl\{ M_{m \nu } \bigl( \vert f \vert ^{m} \bigr) ( y ) \bigr\} ^{\frac{1}{m}}, \end{aligned}$$

where $q_{2}<\tilde{m}<m$.

Finally, we consider $J_{3}$.

*Case of* $q_{1} \geq D$: By Lemmas 9 and 6, we have

26 $$\begin{aligned} & \biggl( \int_{2^{k}r\leq d(x_{0},z)< 2^{k+1}r}\bigl\vert \tilde{\mathcal{K}}(y,z)- \tilde{ \mathcal{K}}(x_{0},z)\bigr\vert ^{m^{\prime}}\,\mathrm{d}z \biggr) ^{\frac{1}{m ^{\prime}}} \biggl( \int_{2^{k}r\leq d(x_{0},z)< 2^{k+1}r}\bigl\vert h(z)-h _{2B}\bigr\vert ^{s^{\prime}}\,\mathrm{d}z \biggr) ^{\frac{1}{s^{\prime}}} \\ & \leq \frac{C_{N}r^{\delta }}{ ( 1+2^{k}r/\rho (x_{0}) ) ^{N}} \biggl( \int_{2^{k}r\leq d(x_{0},z)< 2^{k+1}r}\frac{1}{d ^{m^{\prime}\delta }(x_{0},z)V^{m^{\prime}}(d(x_{0},z))}\,\mathrm{d}z \biggr) ^{\frac{1}{m^{\prime}}} \\ & \quad \times \biggl( \int_{d(x_{0},z)< 2^{k+1}r}\bigl\vert h(z)-h _{2B}\bigr\vert ^{s^{\prime}}\,\mathrm{d}z \biggr) ^{\frac{1}{s^{\prime}}} \\ & \leq \frac{C_{N}}{ ( 1+2^{k}r/\rho (x_{0}) ) ^{N}}\frac{r ^{\delta }}{(2^{k}r)^{\delta }V^{(m^{\prime}-1)/m}(2^{k}r)} \Vert h\Vert _{\operatorname{Lip}_{\nu }^{\theta }(G)}2^{k\nu }r^{\nu } \biggl( 1+\frac{2^{k}r}{ \rho (x_{0})} \biggr) ^{(l_{0}+1)\theta } V^{\frac{1}{s}}\bigl(2^{k}r\bigr) \\ & \leq \Vert h\Vert _{\operatorname{Lip}_{\nu }^{\theta }(G)}2^{k\nu }r^{\nu } \frac{C _{N}}{ ( 1+2^{k}r/\rho (x_{0}) ) ^{N-(l_{0}+1)\theta }} \frac{r ^{\delta }}{(2^{k}r)^{\delta }V^{1/m}(2^{k}r)}, \end{aligned}$$

where $\frac{1}{m}+\frac{1}{m^{\prime}}+\frac{1}{s}=1$ and $\frac{1}{s^{\prime}}+\frac{1}{s}=1$. Therefore, via the Hölder inequality,

$$\begin{aligned} J_{3} &= \frac{1}{\vert B \vert } \int_{B} \bigl\vert A_{3}f(y)-c \bigr\vert \, \mathrm{d}y \\ & \leq \frac{1}{\vert B \vert } \int_{B} \biggl\vert \int_{d(x_{0},z)>2r} \bigl(\tilde{\mathcal{K}}(y,z)-\tilde{ \mathcal{K}}(x_{0},z)\bigr) \bigl(h(z)-h_{2Q}\bigr)f(z) \, \mathrm{d}z\biggr\vert \,\mathrm{d}y \\ & \leq \frac{1}{\vert B \vert } \int_{B} \sum_{k=1}^{\infty } \int _{2^{k}r\leq d(x_{0},z)< 2^{k+1}r} \bigl\vert \bigl(\tilde{\mathcal{K}}(y,z)- \tilde{\mathcal{K}}(x_{0},z)\bigr) \bigl(h(z)-h_{2Q} \bigr)f(z)\bigr\vert \,\mathrm{d}z\, \mathrm{d}y \\ & \leq \frac{\Vert h\Vert _{\operatorname{Lip}_{\nu }^{\theta }(G)}}{\vert B \vert } \int _{B} \sum_{k=1}^{\infty }2^{k\nu }r^{\nu } \frac{C}{ ( 1+2^{k}r/ \rho (x_{0}) ) ^{N-(l_{0}+1)\theta }} \frac{r^{\delta }}{(2^{k}r)^{ \delta }V^{\frac{1}{m}-\frac{1}{s}}(2^{k}r)} \\ & \quad \times \biggl( \int_{d(x_{0},z)< 2^{k+1}r}\bigl\vert f(z) \bigr\vert ^{m}\, \mathrm{d}z \biggr) ^{\frac{1}{m}} \\ & \leq C \Vert h\Vert _{\operatorname{Lip}_{\nu }^{\theta }(G)} \sum_{k=1}^{ \infty } \frac{C_{N}}{ ( 1+2^{k}r/\rho (x_{0}) ) ^{N-(l_{0}+1) \theta }} \frac{r^{\delta }}{(2^{k}r)^{\delta }V^{1/m}(2^{k}r)}\frac{(2^{k}r)^{ \nu }}{(2^{k+1}r)^{\nu }}V^{\frac{1}{m}} \bigl(2^{k+1}r\bigr) \\ & \quad \times \biggl( \frac{(2^{k+1}r)^{m\nu }}{V(2^{k+1}r)} \int _{d(x_{0},z)< 2^{k+1}r}\bigl\vert f(z) \bigr\vert ^{m}\, \mathrm{d}z \biggr) ^{ \frac{1}{m}} \\ & \leq C \Vert h\Vert _{\operatorname{Lip}_{\nu }^{\theta }(G)} \sum_{k=1}^{ \infty }2^{-k\delta } \frac{C_{N}}{ ( 1+2^{k}r/\rho (x_{0}) ) ^{N-(l_{0}+1)\theta }} \bigl\{ M_{m\nu } \bigl( \vert f \vert ^{m} \bigr) ( y ) \bigr\} ^{\frac{1}{m}} \\ & \leq C \Vert h\Vert _{\operatorname{Lip}_{\nu }^{\theta }(G)} \bigl\{ M_{m \nu } \bigl( \vert f \vert ^{m} \bigr) ( y ) \bigr\} ^{\frac{1}{m}} \end{aligned}$$

if we choose *N* sufficiently large.

*Case of* $\frac{D}{2} \leq q_{1} < D$: By Lemmas 9 and 6, we have

$$\begin{aligned} & \biggl( \int_{2^{k}r\leq d(x_{0},z)< 2^{k+1}r}\bigl\vert \tilde{\mathcal{K}}(y,z)- \tilde{ \mathcal{K}}(x_{0},z)\bigr\vert ^{p}\,\mathrm{d}z \biggr) ^{ \frac{1}{p}} \biggl( \int_{2^{k}r\leq d(x_{0},z)< 2^{k+1}r}\bigl\vert h(z)-h _{2B}\bigr\vert ^{t}\,\mathrm{d}z \biggr) ^{\frac{1}{t}} \\ & \quad \leq \frac{C_{N}r^{\delta }(2^{k}r)^{1-\delta }}{V(2^{k}r) ( 1+2^{k}r/ \rho (x_{0}) ) ^{N}} \biggl( \int _{2^{k}r\leq d(x_{0},z)< 2^{k+1}r}\biggl\vert \int_{B(z,2^{k+3}r)} \frac{d(z,y)W(y)}{V(z,y)} \,\mathrm{d}y\biggr\vert ^{p}\,\mathrm{d}z \biggr) ^{\frac{1}{p}} \\ & \quad \quad {}\times \biggl( \int_{2^{k}r\leq d(x_{0},z)< 2^{k+1}r} \bigl\vert h(z)-h_{2B}\bigr\vert ^{t}\,\mathrm{d}z \biggr) ^{\frac{1}{t}} \\ &\quad \quad {}+ \frac{C _{N}\Vert h\Vert _{\operatorname{Lip}_{\nu }^{\theta }(G)}2^{l\nu }r^{\nu }}{ ( 1+2^{k}r/\rho (x_{0}) ) ^{N-(l_{0}+1)\theta }} \frac{r^{ \delta }}{(2^{k}r)^{\delta }V^{1/m}(2^{k}r)} \\ & \quad \leq \frac{C_{N}\Vert h\Vert _{\operatorname{Lip}_{\nu }^{\theta }(G)}2^{l \nu }r^{\nu }}{ ( 1+2^{k}r/\rho (x_{0}) ) ^{N-(l_{0}+1)\theta }} \\ & \quad \quad {}\times \biggl[ \frac{r^{\delta }(2^{k}r)^{1-\delta }}{V^{ \frac{t-1}{t}}(2^{k}r)} \biggl( \int_{B(x_{0},2^{k+3}r)}W^{q_{1}}(z) \,\mathrm{d}z \biggr) ^{\frac{1}{q_{1}}}+\frac{r^{\delta }}{(2^{k}r)^{ \delta }V^{1/m}(2^{k}r)} \biggr] \\ & \quad \leq \frac{C_{N}\Vert h\Vert _{\operatorname{Lip}_{\nu }^{\theta }(G)}2^{l \nu }r^{\nu }}{ ( 1+2^{k}r/\rho (x_{0}) ) ^{N-(l_{0}+1)\theta }} \\ & \quad \quad{} \times \biggl[ \frac{r^{\delta }V^{\frac{1}{q_{1}}}(2^{k}r)(2^{k}r)^{-1- \delta }}{V^{\frac{t-1}{t}}(2^{k}r)} \biggl( \frac{(2^{k+3}r)^{2}}{V(2^{k+3}r)} \int_{B(x_{0},2^{k+3}r)}W(z) \,\mathrm{d}z \biggr) +\frac{r^{ \delta }}{(2^{k}r)^{\delta }V^{1/m}(2^{k}r)} \biggr] \\ & \quad \leq \frac{C_{N}\Vert h\Vert _{\operatorname{Lip}_{\nu }^{\theta }(G)}2^{l \nu }r^{\nu }}{ ( 1+2^{k}r/\rho (x_{0}) ) ^{N-l_{1}-(l_{0}+1) \theta }} \biggl( \frac{r^{\delta }V^{\frac{1}{q_{1}}}(2^{k}r)(2^{k}r)^{-1- \delta }}{V^{\frac{t-1}{t}}(2^{k}r)} +\frac{r^{\delta }}{(2^{k}r)^{ \delta }V^{1/m}(2^{k}r)} \biggr) \\ & \quad \leq \frac{C\Vert h\Vert _{\operatorname{Lip}_{\nu }^{\theta }(G)}2^{l\nu }r ^{\nu }}{ ( 1+2^{k}r/\rho (x_{0}) ) ^{N-l_{1}-(l_{0}+1)\theta }} \frac{r^{\delta }}{(2^{k}r)^{\delta }V^{1/m}(2^{k}r)}, \end{aligned}$$

where $\frac{1}{m}+\frac{1}{p}+\frac{1}{t}=1$ and $\frac{1}{q_{1}}= \frac{1}{p}+\frac{1}{D}$. Therefore, for $m>q_{2}$,

$$\begin{aligned} J_{3} &= \frac{1}{\vert B \vert } \int_{B} \bigl\vert A_{3}f(y)-c \bigr\vert \, \mathrm{d}y \\ & \leq \frac{1}{\vert B \vert } \int_{B} \biggl\vert \int_{d(x_{0},z)>2r} \bigl(\tilde{\mathcal{K}}(y,z)-\tilde{ \mathcal{K}}(x_{0},z)\bigr) \bigl(h(z)-h_{2Q}\bigr)f(z) \, \mathrm{d}z\biggr\vert \,\mathrm{d}y \\ & \leq \frac{1}{\vert B \vert } \int_{B} \sum_{k=1}^{\infty } \int _{2^{k}r\leq d(x_{0},z)< 2^{k+1}r} \bigl\vert \bigl(\tilde{\mathcal{K}}(y,z)- \tilde{\mathcal{K}}(x_{0},z)\bigr) \bigl(h(z)-h_{2Q} \bigr)f(z)\bigr\vert \,\mathrm{d}z\, \mathrm{d}y \\ & \leq \frac{\Vert h\Vert _{\operatorname{Lip}_{\nu }^{\theta }(G)}}{\vert B \vert } \int _{B} \sum_{k=1}^{\infty }2^{k\nu }r^{\nu } \frac{C}{ ( 1+2^{k}r/ \rho (x_{0}) ) ^{N-l_{1}-(l_{0}+1)\theta }} \frac{r^{\delta }}{(2^{k}r)^{ \delta }V^{\frac{1}{m}-\frac{1}{s}}(2^{k}r)} \\ & \quad \times \biggl( \int_{d(x_{0},z)< 2^{k+1}r}\bigl\vert f(z) \bigr\vert ^{m}\, \mathrm{d}z \biggr) ^{\frac{1}{m}} \\ & \leq C \Vert h\Vert _{\operatorname{Lip}_{\nu }^{\theta }(G)} \sum_{k=1}^{ \infty } \frac{C_{N}}{ ( 1+2^{k}r/\rho (x_{0}) ) ^{N-l_{1}-(l _{0}+1)\theta }} \frac{r^{\delta }}{(2^{k}r)^{\delta }V^{1/m}(2^{k}r)}\frac{(2^{k}r)^{ \nu }}{(2^{k+1}r)^{\nu }}V^{\frac{1}{m}} \bigl(2^{k+1}r\bigr) \\ & \quad \times \biggl( \frac{(2^{k+1}r)^{m\nu }}{V(2^{k+1}r)} \int _{d(x_{0},z)< 2^{k+1}r}\bigl\vert f(z) \bigr\vert ^{m}\, \mathrm{d}z \biggr) ^{ \frac{1}{m}} \\ & \leq C \Vert h\Vert _{\operatorname{Lip}_{\nu }^{\theta }(G)} \sum_{k=1}^{ \infty }2^{-k\delta } \frac{C_{N}}{ ( 1+2^{k}r/\rho (x_{0}) ) ^{N-l_{1}-(l_{0}+1)\theta }} \bigl\{ M_{m\nu } \bigl( \vert f \vert ^{m} \bigr) ( y ) \bigr\} ^{\frac{1}{m}} \\ & \leq C \Vert h\Vert _{\operatorname{Lip}_{\nu }^{\theta }(G)} \bigl\{ M_{m \nu } \bigl( \vert f \vert ^{m} \bigr) ( y ) \bigr\} ^{\frac{1}{m}}, \end{aligned}$$

if we choose *N* sufficiently large. □

## Proof of the main result

### Proof of Theorem 1

Suppose $h \in \operatorname{Lip}_{\nu }^{\theta }(G)$. We choose *m* such that it satisfies $q_{2} < m < p$. We conclude from Proposition 4, Lemmas 5, 11 and 12 that

$$\begin{aligned} \bigl\Vert [h,\tilde{\mathcal{R}}]f\bigr\Vert _{L^{p}(G)}^{p} &\leq\int _{G}\bigl\vert M_{\rho ,\alpha }\bigl([h,\tilde{ \mathcal{R}}]f\bigr) (x)\bigr\vert ^{p} \,\mathrm{d}x \\ &\leq C\int_{G}\bigl\vert M_{\rho ,\beta }^{\sharp } \bigl([h, \tilde{\mathcal{R}}]f\bigr) (x)\bigr\vert ^{p}\,\mathrm{d}x \\ &\quad {}+C\sum_{k}\vert Q _{k} \vert \biggl( \frac{1}{\vert Q_{k}\vert } \int_{2Q_{k}} \bigl\vert [h,\tilde{\mathcal{R}}]f(x)\bigr\vert \,\mathrm{d}x \biggr) ^{p} \\ &\leq C \int_{G}\bigl\vert M_{\rho ,\beta }^{\sharp } \bigl([h, \tilde{\mathcal{R}}]f\bigr) (x)\bigr\vert ^{p}\, \mathrm{d}x \\ &\quad {}+C\Vert h\Vert _{\operatorname{Lip}_{\nu }^{\theta }(G)}^{p}\sum _{k} \int_{2Q_{k}}\bigl\vert \bigl\{ M_{m \nu }\bigl(\vert f \vert ^{m}\bigr)\bigr\} ^{\frac{1}{m}}(x)\bigr\vert ^{p} \, \mathrm{d}x \\ &\leq C\Vert h\Vert _{\operatorname{Lip}_{\nu }^{\theta }(G)}\bigl\Vert \bigl(M_{m\nu } \bigl( \vert f\vert ^{m}\bigr)\bigr)^{\frac{1}{m}}\bigr\Vert _{L^{p}(G)}^{p} \\ &\leq C\Vert h\Vert _{\operatorname{Lip}_{\nu }^{\theta }(G)}\Vert f\Vert _{L^{q}(G)}^{p}, \end{aligned}$$

where $\frac{1}{p}=\frac{1}{q}-\frac{\nu }{D}$. □

## Conclusions

We prove the $L^{p}\rightarrow L^{q}$ boundedness for the commutator which is generated by the Riesz transform $\mathcal{R} $ and the function $h\in \operatorname{Lip}^{\theta }_{\nu }$. We generalize the corresponding results on the Euclidean space in [6] to the nilpotent Lie group, and they may have some applications in harmonic analysis and PDE on the Lie group.
